# Bacteriophage-Fused Peptides for Serodiagnosis of Human Strongyloidiasis

**DOI:** 10.1371/journal.pntd.0002792

**Published:** 2014-05-29

**Authors:** Nágilla Daliane Feliciano, Vanessa da Silva Ribeiro, Fabiana de Almeida Araújo Santos, Patricia Tiemi Fujimura, Henrique Tomaz Gonzaga, Luiz Ricardo Goulart, Julia Maria Costa-Cruz

**Affiliations:** 1 Laboratório de Diagnóstico de Parasitoses, Instituto de Ciências Biomédicas, Universidade Federal de Uberlândia, Uberlândia, Minas Gerais, Brasil; 2 Laboratório de Nanobiotecnologia, Instituto de Genética e Bioquímica, Universidade Federal de Uberlândia, Uberlândia, Minas Gerais, Brasil; 3 Department of Medical Microbiology and Immunology, University of California, Davis, Davis, California, United States of America; George Washington University, United States of America

## Abstract

**Background:**

Strongyloidiasis, a human intestinal infection caused by the nematode *Strongyloides stercoralis*, is frequently underdiagnosed and although its high prevalence is still a neglected parasitic disease because conventional diagnostic tests based on parasitological examination (presence of *Strongyloides* larvae in stool) are not sufficiently sensitive due to the low parasitic load and to the irregular larval output. There is an urgent need to improve diagnostic assays, especially for immunocompromised patients with high parasitic load as consequence of self-infection cycle, which can disseminate throughout the body, resulting in a potentially fatal hyperinfection syndrome often accompanied by sepsis or meningitis.

**Methods/Principal Findings:**

We have performed Phage Display technology to select peptides that mimic *S. stercoralis* antigens, capable of detecting a humoral response in patients with strongyloidiasis. The peptides reactivity was investigated by Phage-ELISA through different panels of serum samples. We have successfully selected five peptides with significant immunoreactivity to circulating IgG from patients' sera with strongyloidiasis. The phage displayed peptides C9 and C10 presented the highest diagnostic potential (AUC>0.87) with excellent sensitivity (>85%) and good specificity (>77.5%), suggesting that some *S. stercoralis* antigens trigger systemic immune response.

**Conclusions/Significance:**

These novel antigens are interesting serum biomarkers for routine strongyloidiasis screenings due to the easy production and simple assay using Phage-ELISA. Such markers may also present a promising application for therapeutic monitoring.

## Author Summary

Strongyloidiasis is one of the most neglected helminthic infections and can cause disseminated disease in immunocompromised hosts, which can be fatal. Given the unsatisfactory results of current parasitological and serological tests, there is a need for more efficient diagnostic tools. Therefore we have used phage display technology and bioppaning procedure to select sensitive and specific mimotopes ready to be used in immunodiagnostic tests. These mimotopes allows a cheap and fast clear-cut diagnosis of *Strongyloides stercoralis* infections. The field applicability of the assay using the phage clones obtained is really promising. The main advantage is that phage-based ELISA is the reproducible, simple, rapid and low-cost for production of recombinant antigens, and such tests may be of interest for massive screening in developing countries. Our results indicate that the mimotopes selected and tested here are potential biomarkers for the diagnosis of human strongyloidiasis.

## Introduction

Human strongyloidiasis is a parasitic disease caused by the nematode *Strongyloides stercoralis*. It is one of the major intestinal infections in humans with worldwide distribution, and affects 50–100 million people in 70 countries, mainly in tropical and subtropical regions and also in some areas of Europe and Asia [1]–[3] Strongyloidiasis is a public health problem, and although its high prevalence is still a neglected parasitic disease in many countries. It is believed that the number of individuals infected with *S. stercoralis* can be more frequent than expected [3], [4].

The clinical presentation of human strongyloidiasis varies with the status of host immunity. *S. stercoralis* causes chronic asymptomatic infections of the gastrointestinal tract in immunocompetent human hosts and may remain undetected for long periods [5], [6]. In contrast, immunocompromised hosts present systemic invasion of the parasite that may develop into hyperinfection syndrome or disseminated strongyloidiasis fatal forms [7]–[10].

Definitive diagnosis of *S. stercoralis* infection relies mainly on demonstration of larval stages in fecal specimes. However, since majority of cases involves low and irregular larval output, negative parasitological results cannot be interpreted as absence of infection [1], [11]–[13]. Indirect diagnosis based on serology is widely used in addition to the parasitological analysis of stool specimens [14]–[18].

A limitation in strongyloidiasis immunodiagnosis has been attributed to the difficulty of obtaining sufficient quantities of *S. stercoralis* larvae to prepare antigenic extracts used in these assays, and also to the cross-reactivity in sera from patients with other helminthic infections. New and promising tools such as serological methods based on recombinant antigens and molecular-based techniques are also available in some referral centers [19]–[24]. Recently, serodiagnosis of strongyloidiasis has been achieved with excellent sensitivity and specificity using a 31-kDa recombinant antigen termed NIE [25], [26] and a luciferase immunoprecipitation system; however, these recombinant antigens require specific luciferase-antigen fusion construct and mammalian cell cultures, which is not easy to be attained, and is not available for simple laboratory settings. Due to difficulties to product crude extracts and some other recombinant antigens our aim was to develop a strategy for simpler antigen production by Phage Display to detect IgG in large population screenings.

The Phage Display technology has been widely used for numerous purposes, as to map protein–ligand interactions, identify binding antagonists and enzyme inhibitors through the design of mimotopes, and also employed to select epitope-mimicking antigens and immunogens, which have served as the basis for development of diagnostic platforms and novel vaccines [27]–[30]. Selected mimotopes may be used in a simple, specific and low cost phage ELISA as described by several authors [31]–[35]. The aim of this study was to develop novel antigens for serodiagnosis of *S. stercoralis* with improved sensitivity and specificity, and reduced cross-reactions with other parasite infections, a major problem when crude antigens are used. This is the first and successful application of Phage Display to identify and validate potential mimotopes of *S. stercoralis*, which can be used to detect circulating IgG in human strongyloidiasis diagnosis.

## Materials and Methods

### Ethical considerations and patients recruitment

The study was approved in 2009 by the Research Ethics Committee from Federal University of Uberlândia (CEP-UFU; number 553/09), State of Minas Gerais, Brazil. Serum samples were obtained at the Clinics' Hospital of the Federal University of Uberlândia (UFU), State of Minas Gerais, Brazil. All samples were analysed anonymously by two independent technical experts in parasitological analysis, and serological tests for new antigens were performed in 2012–2013.

### Serum samples

This is a retrospective study that used a panel of 120 serum samples divided into three groups. Group I consisted of 40 patients living in an endemic area and with confirmed serological and parasitological diagnosis of strongyloidiasis using Baermann and Moraes methods, based on positive larval thermo-hydrotropism and Lutz method, a gravity sedimentation technique [36]–[38], as reference standard. Group II included 40 patients with positive diagnosis of other parasites including *Ascaris lumbricoides* (n = 7), *Enterobius vermicularis* (n = 5), *Schistosoma mansoni* (n = 3), *Hymenolepis nana* (n = 7), *Taenia* sp. (n = 5), hookworm (n = 8), *Trichuris trichiura* (n = 2) and *Giardia lamblia* (n = 3). The samples from Groups I and II were obtained from patients affected by single infection. Group III contained 40 apparently healthy individuals based on their clinical observation, without evidence of contact with *S. stercoralis* infection or previous history of strongyloidiasis and three fecal samples tested negative. Serum samples were obtained at the Clinics' Hospital of the Federal University of Uberlândia (UFU), State of Minas Gerais, Brazil.

### Immunoglobulin G purification

Immunoglobulin G was purified from a different panel of serum samples to be used in ELISA assays. This panel consisted of six pooled samples of each group. Purification was performed by protein G-coupled magnetic beads (2–8 µm diameter), according to the manufacturer's recommendation (Dynabeads IgG, Invitrogen). Antibody quantity was estimated as described [39], where: sample concentration  =  absorbance at 280 nm×dilution factor/extinction coefficient at 280 nm (1.36 for IgG).

### Saline extract from *S. venezuelensis*

For antigenic extraction, 300.000 *S. venezuelensis* filariform larvae were resuspended in phosphate-buffered saline (PBS, 0.01 mol/L, pH 7.2) and disrupted in an ice bath using a tissue homogenizer (OMNI International, Kennesaw, USA) with 5 cycles of 5 min each, and then submitted to 8 ultrasound cycles for 20 s at 40 kHz (Thorton, Inspec Eletrônica, São Paulo, Brazil). After an overnight incubation period at 4°C under constant gentle shaking, the suspension was centrifuged at 12 400 g for 30 min at 4°C and the supernatant (saline extract) was quantified [40], and aliquots were subdivided and stored at −20°C until use on competitive Phage-ELISA.

### Phage display peptide library

The Ph.D.-C7C Phage Display Peptide Library Kit (New England Biolabs, Ipswich, USA) based on a 7-mer random peptide combinatorial library fused to the pIII capsid of the M13 phage was used for ligands' selection. The wild-type M13 phage, from which the peptide library was derived, was used as negative control.

### Biopanning

The Ph.D.-C7C library was submitted to negative and positive selections against purified IgGs from a pool of individuals from Groups I, II and III. The biopanning procedure was performed as previously described [41] with modifications. We used 10 µL (1×10^11^ phages) of Ph.D.-C7C library diluted in 190 µL TBS-T (TBS containing 0.1% polysorbate 20; Tris-buffered saline (TBS): 137 mM NaCl, 5.1 mM KCl, 1.35 mM CaCl_2_, 1.05 mM MgCl_2_, 1.4 mM Na_2_HPO_4_, 24.8 mM Tris, pH 7.5). Selection was performed using 20 µl from purified IgG from each group, immobilized (100 µg/mL in 0.1 M NaHCO_3_, pH 8.6) on polystyrene microplates (Nunc Maxisorp). For negative selection, the library was incubated with purified IgG from healthy individuals (Group III; 30 min at room temperature (RT); and next, supernatant with non-bound phages was added to IgG from patients with other parasites (Group II; 30 min at RT). After this second subtraction step, a positive selection was performed by using non-bound phages that were incubated with purified IgG from patients with definitive diagnosis of strongyloidiasis under the same conditions. Non-bound phages were removed after 10 washings, using TBS-T 0.01% in the first round, and TBS-T 0.05% in subsequent rounds. Bound-phage particles were eluted using glycine (pH 2.0) and neutralized with Tris (75 µL, pH 9). Small aliquots of the eluted phages were saved for titration. The remaining aliquot was amplified in *Escherichia coli* ER2738 (New England Biolabs) to the next two additional rounds of biopanning to enrich the clones that bind to IgG anti-*Strongyloides*. The eluted phages were amplified and titrated.

### DNA sequencing and bioinformatic analysis

After the third round of biopanning, individual phage clones were randomly selected and amplified for DNA sequencing. Purified single strand phage DNA fragments were extracted and sequenced with 200 ng of primer -96 gIII (5′-OH CCC TCA TAG TTA GCG TAA CG-3′; New England Biolabs, Ipswich, USA) and premix using an automatic capillary sequencer (DYEnamic ET Dye Terminator Kit and MegaBACE 1000 Genetic Analyzer; Amersham Biosciences, Piscataway, USA). Amino acid sequences were deduced according to nucleotide sequences and analyzed using the software ExPASy available on line (http://www.expasy.org). The alignment among the sequences was performed by Clustal Omega program (http://www.ebi.ac.uk/Tools/msa/clustalo/), and the similarity with proteins from *S. stercoralis* was verified using BLASTp search (http://www.ncbi.nlm.nih.gov/blast).

### Immunoreactivity of the selected phage clones to human IgG in Strongyloidiasis diagnosis by Phage-ELISA

Phage-ELISA was performed as described previously [35] with modifications for validation of selected phage clones. Preliminary experiments were conducted to determine the optimal conditions for ELISA by titrating reagents (phages, sera and conjugate). Briefly, polystyrene microplates (Nunc MaxiSorp) were coated with 1×10^10^ plaque-forming unit (PFU) of each one of the five selected phage clones (B2, B4, C9, C10 and D3) in carbonate bicarbonate buffer (pH 9.6, overnight, 4°C). Microplates were washed once with PBS containing 0.05% polysorbate 20 (PBS-T) and blocked with 200 µL of skimmed milk 3% in PBS-T (PBS-TM) at 37°C for 60 min. PBS-TM was used as the dilution solution for serum and conjugate. Next, 50 µL/well of serum samples (1∶80) were added and incubated (60 min, 37°C). After incubation, plates were washed three times with PBS-T, enzyme conjugate (goat anti-human IgG-peroxidase, Fc specific Sigma, St. Louis, USA) was added 1∶2000 in PBS-TM and incubated for 60 min at 37°C. The assay was developed, after another washing procedure, by the addition of hydrogen peroxide and orthophenylenediamine (OPD) in 0.1 M citrate phosphate Na_2_HPO_4_ buffer pH 5.5 for 15 min. The reaction was interrupted with H_2_SO_4_ (2 N).

Optical densities (OD) were determined at 492 nm in an ELISA reader (TP-Reader ThermoPlate). All samples were tested in duplicates. Each serum sample was tested against wild-type M13 phage as negative control. The final OD was adjusted by the ratio of OD readings for selected phage clones to the OD of the wild-type M13 phage. The optimum point of reaction (cut-off) for each phage clone was determined using the receiver operating characteristic (ROC curve) using GraphPad software package 5.0 (GraphPad Software Inc., San Diego, USA). The reactivity index (RI) was calculated based on the cut-off point (RI  =  adjusted OD of each sample/cut-off). Samples with RI> 1 were considered positive.

### Competitive Phage-ELISA assays

Competitive ELISA was used to analyze the binding specificities of phage clones to the pool of sera from patients with strongyloidiasis. First, pooled sera diluted 1∶80 in PBS-TM were incubated with serial dilutions of saline extract from *S. venezuelensis* (1, 5, 12.5 and 25 µg/mL) in microtubes in a final volume of 100 µL and incubated at 37°C for 2 h. Then, 100 µL from each microtube were transferred to polystyrene microplate wells previously coated with phage clones or wild-type M13 phage (1×10^10 ^pfu/mL), plates were blocked with PBS-TM and incubated for 60 min at 37°C. The following steps were performed as described earlier for Phage-ELISA, using peroxidase-goat anti-human IgG, Fc specific (Sigma, St. Louis, USA) diluted 1∶2000.

### Statistical analysis

Data were analyzed by using the GraphPad software package 5.0 (GraphPad Software Inc., San Diego, USA) to build receiver operating characteristic curves (ROC) and template for two-graph ROC (TG-ROC) analysis [42]. ROC and TG-ROC analysis were combined to define the best cut-off value and to describe diagnostic parameters. Area under ROC curve (AUC) was calculated [43] and compared using Hanley and McNeil method [44]. Sensitivity (Se), specificity (Sp), diagnostic efficiency (DE) and likelihood ratios (LR+ e LR−) were obtained from ROC tables. Probability (*P*) values of<0.05 were regarded as significant and 95% confidence intervals (CI 95%) were provided. The Friedman statistic with Dunn post hoc was used to determine differences among groups for phage clone reactivity.

## Results

Phage display selections were performed in 2010 and 2011, and antigen validations were conducted in 2012 and 2013 with retrospective serum samples collected in 2009 in the Reference Laboratory for Parasitological Diagnosis at the Federal University of Uberlandia. Adult patients (18–84 years of age) of both genders were recruited from the Clinical Hospital, and only those with single infection were considered for this study, and stratified according to their parasite infection.

### Biopanning and bioinformatic analyses

After three rounds of biopanning, fifty phage clones were randomly selected to determine their DNA sequences and their translated peptides. Among them, fourteen different clones were characterized. In an attempt to identify regions that contain similar amino acid residues, the selected peptides were aligned using the Clustal Omega software, and no consensus motif was obtained among different sequences. In our BLAST search to determine linear similarity with proteins of interest from *S. stercoralis*, we have shown significant similarity of five phage clones with *S. stercoralis* proteins (table 1).

**Table 1:** Selected mimotopes aligned with putative proteins of *S. stercoralis*.

Mimotope	Putative protein (amino acid positions) (access number genebank)	
B2	1)	nuclear hormone receptor of the steroid/thyroid hormone receptors superfamily [*Strongyloides stercoralis*] (489–492) (AAD37372.1)
	2)	IgG-immunoreactive zinc finger protein [*Strongyloides stercoralis*] (49–52) (AAF04101.1)
	3)	SMAD-1 [*Strongyloides stercoralis*] (69–73) (AGC25442.1)
B4	1)	aspartic protease precursor [*Strongyloides stercoralis*] (121–129) (AAD09345.1)
	2)	DNAJ-like protein [*Strongyloides stercoralis*] (4–9) (AAF22128.1)
	3)	NADH dehydrogenase subunit 4 [*Strongyloides stercoralis*] (342–346) (CAD90563.1)
	4)	forkhead transcription factor 1 isoform a [*Strongyloides stercoralis*] (144–148) (AAQ23177.1)
	5)	TPA_inf: eukaryotic translation elongation factor 1A [*Strongyloides stercoralis*] (191–195) (DAA05873.1)
	6)	NADH dehydrogenase subunit 1 [*Strongyloides stercoralis*] (238–241) (CAD90556.1)
	7)	phosphatidylinositol 3-kinase catalytic subunit [*Strongyloides stercoralis*] (589–593) (AFJ11261.1)
C9	1)	forkhead transcription factor 1 isoform a [*Strongyloides stercoralis*] (180–185) (AAQ23177.1)
C10	1)	phosphatidylinositol 3-kinase catalytic subunit [*Strongyloides stercoralis*] (1202–1205) (AFJ11261.1)
	2)	insulin-like receptor protein tyrosine kinase isoform A [*Strongyloides stercoralis*] (1295–1299) (AGC25443.1)
	3)	insulin-like receptor protein tyrosine kinase isoform B [*Strongyloides stercoralis*] (1333–1337) (AGC25444.1)
	4)	cytochrome c oxidase subunit I [*Strongyloides stercoralis*] (498–501) (CAD90560.1)
D3	1)	nuclear hormone receptor of the steroid/thyroid hormone receptors superfamily [*Strongyloides stercoralis*] (84–87) (AAD37372.1)
	2)	SMAD-1 [*Strongyloides stercoralis*] (172–176) (AGC25442.1)
	3)	cytochrome oxidase subunit 1 [*Strongyloides stercoralis*] (174–180, 146-147) (CAD90560.1)
	4)	cytochrome oxidase subunit 1 [*Strongyloides stercoralis*] (84–90, 56–57) (BAJ22590.1)
	5)	cytochrome c oxidase subunit I, partial (mitochondrion) [*Strongyloides stercoralis*] (159–165,131–132) (AAB71721.1)

### Immunoreactivity of the selected phage clones to human IgG in strongyloidiasis diagnosis by Phage-ELISA

**Figure 1:**
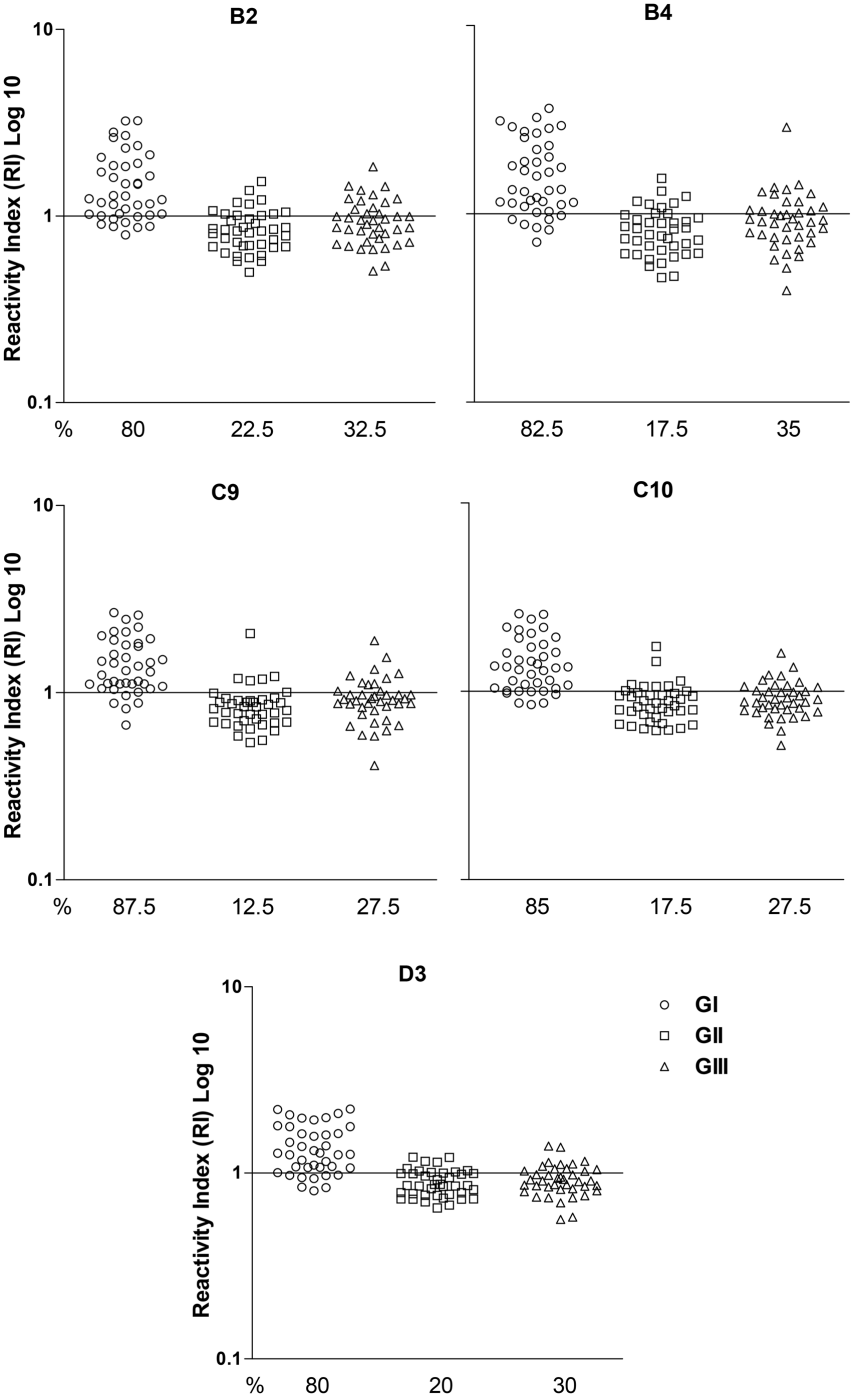
Detection of immunoglobulin G antibodies anti- *S. stercoralis* by phage-ELISA. The assay was performed in serum samples of patients with a definitive diagnosis of strongyloidiasis (Group I; n = 40), other parasites (Group II; n = 40) and apparently healthy individuals (Group III; n = 40) using the phage clones B2, B4, C9, C10 and D3. The horizontal bar indicates the cut off (Reactivity Index - RI = 1); % = positivity.

**Figure 2:**
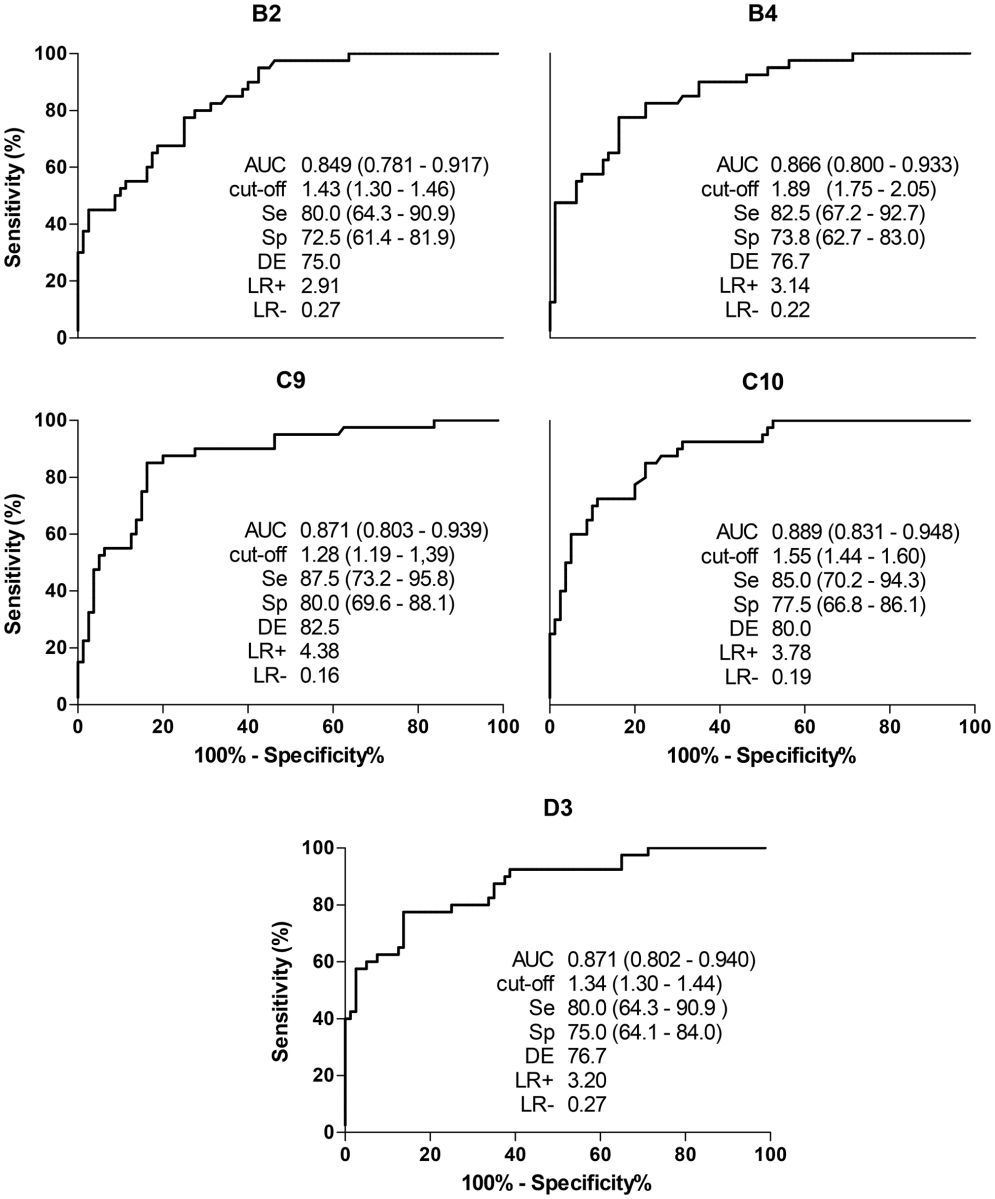
ROC curve analyses of IgG detection in serum samples using phage-clones. The figure shows AUC (area under curve), cut-off, sensitivity (Se), specificity (Sp), diagnostic efficiency (DE) and LR (likelihood ratios) in the optimum point of reaction for the phage clones B2, B4, C9, C10 and D3. (95% confidence interval).

To investigate sensitivity and cross-reactivity of the phage clones against circulating IgG in serum samples from the three groups, ELISA tests were performed. Figure 1 shows the levels of IgG anti-*S. stercoralis* expressed in RI for the serum samples from Groups I, II, and III. Precisely 87.5% of the serum samples from Group I were positive for phage clones C9, whereas for the phage clones C10, B4, D3 and B2, the positivity in this group was respectively, 85%, 82.5%, 80%, and 80%. In Group II, 22.5% of the serum samples were positive for phage clone B2, but only 12.5% were positive for the phage clone C9. In Group III when testing phage clones B4, B2, and D3 the positivity represented, respectively, 35%, 32.5% and 30%; while C9 and C10 showed 27.5% of positivity. ROC curve with area under curve (AUC), Se, Sp, DE and LRs are represented in figure 2. The cutoff value for B2 phage clone for IgG recognition in serum resulted in Se of 80%, Sp of 72.5%, and DE of 75% presenting, at this point, a lower performance; the diagnostic efficiency for the phage clones C10, B4 and D3 were respectively 80%, 76.7% and 76.7%. C9 demonstrated the greatest diagnostic performance considering Sp, Se and DE of 87.5%, 80%, and 82.5%, respectively. The AUC was greater than 0.8 for all phage clones, ranging from 0.849 (B2) to 0.889 (C10). No statistical difference was observed among ELISA results (*P>*0.05). Likelihood ratios showed better values for C9 (LR+ = 4.38, LR− = 0.16). Cross-reactivity of the phage clones with serum samples from patients with other parasites is showed in table 2. All clones reacted with at least one serum sample from patients with *A. lumbricoides* and *E. vermicularis* tested; otherwise sera from *Taenia* sp. patients were not detected. C9 was the only clone that did not cross-react with hookworm patient sera and recognized few other species of parasites sampled. When we compared the difference of reactivity among the groups (GI, GII or GIII) with tested clones (B2, B4, C9, C10 and D3), only GII showed significant differences (F = 17.46, *P* = 0.0016). Levels detected by clone B4 were lower than those detected by D3 (Dunn = −55, *P*<0.05) in this group. IgG detection levels by all phage clones in GI were larger than GII and GIII (F*>*37.55, p<0.0001), which showed no significant difference among them.

**Table 2:** Cross-reactivity of sera from patients with other parasitic diseases (Group II, n = 40) by Phage-ELISA for detection of immunoglobulin G (IgG) to *S. stercoralis* using the selected phage clones.

	Phage-ELISA, n+ (%)				
Parasite	B2	B4	C9	C10	D3
Hookworm (n = 8)	2 (25)	1 (12.5)	0	1 (12.5)	1 (12.5)
*Ascaris lumbricoides* (n = 7)	2 (28.6)	2 (28.6)	3 (42.8)	2 (28.6)	3 (42.8)
*Enterobius vermicularis* (n = 5)	2 (40)	2 (40)	1 (20)	1 (20)	2 (40)
*Giardia lamblia* (n = 3)	0	0	0	1 (33.3)	1 (33.3)
*Hymenolepis nana* (n = 7)	0	0	0	1 (14.3)	0
*Schistosoma mansoni* (n = 3)	2 (66.6)	1 (33.3)	0	1 (33.3)	0
*Taenia* sp. (n = 5)	0	0	0	0	0
*Trichuris trichiura* (n = 2)	1 (50)	1 (50)	1 (50)	0	1 (50)

### Competitive Phage-ELISA assays

**Figure 3:**
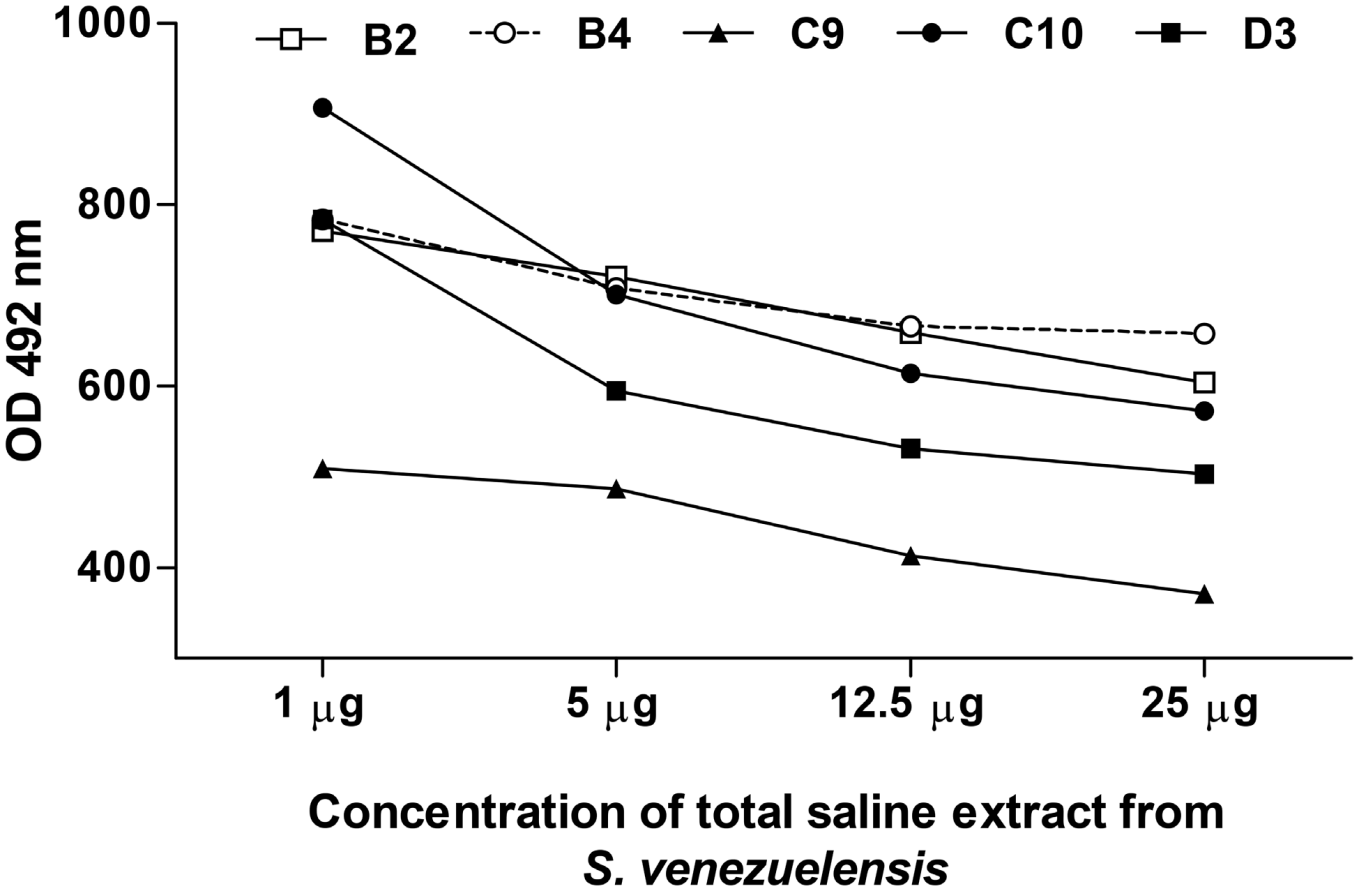
IgG competitive phage-ELISA assay. Plates were coated with each phage clones (1×10^10^ pfu/mL) selected by phage display and serum samples were pre-incubated with increasing amounts (1–25 µg/mL) of saline extract from larvae of *S. venezuelensis.*

To analyze the capacity of peptide to mimic the *S. stercoralis* antigens, a competitive ELISA was performed. Figure 3 shows that each phage clone was partially inhibited in a dose dependent manner when serum of *S. stercoralis* patient was pre-incubated with saline extract from *S. venezuelensis* (heterologous antigen). In contrast, no inhibition of this interaction was observed by the addition of a wild-type phage. These data showed that phage displayed peptides reacted specifically with antibody from patients with Strongyloidiasis demonstrating that there was a competition for IgG between mimotopes and the antigenic extract.

## Discussion

This is the first study that has successfully selected mimotopes by phage display to improve serodiagnosis of human strongyloidiasis. Strongyloidiasis is frequently under diagnosed since many infections remain asymptomatic and conventional diagnostic tests based on parasitological examination are not sufficiently sensitive, due to the absence of elimination of eggs, intermittent larval elimination, and low production of larvae in the chronic stage in asymptomatic patients, making it difficult to detect infection [1], [45], [46]. The immunological detection of specific antibodies against *Strongyloides* is useful for diagnosis because it complements parasitological analysis [14], [16], [17], but has limitations as difficulties to obtain sufficient quantities of *Strongyloides* larvae used in antigenic preparations and also due to cross-reactivity with other helminths. There are no commercial kits available with *S. stercoralis* recombinant antigens. Therefore, the heterologous antigen from *S. venezuelensis* has been used as an alternative, due to antigenic similarity with *S. stercoralis* [18], [47], and because it does not present a risk to human health and can be easily obtained in rats [5]. We have properly demonstrated the utility of *S. venezuelensis* in serodiagnosis of strongyloidiasis [18] with a diagnostic efficiency of 90% using a crude saline extract. However, its use requires maintenance in a large number of rats that should be frequently replaced due to acquired resistance, besides the period to attain the parasite cycle.

The diagnostic difficulties led us to propose phage display as an strategy to develop novel antigens to detect circulating IgGs during *Strongyloides* infections. Selected phage-fused peptides can mimic true epitope regions of *S. stercoralis* antigens, and are referenced as mimotopes. Our strategy was to develop mimotopes with diagnostic potential by evaluating polyclonal sera instead of antibody fractions, and the use of affinity purified IgG against specific protein targets [48], [49], which led us to select clones with fourteen different sequence motifs, suggesting that these are immunodominant epitopes that are present in major proteins that have triggered a humoral response during infection. From these, five phage clones were selected based on their higher reactivity in Phage-ELISA tests, and on their potential to discriminate positive from negative sera samples.

All phage clones showed ability to distinguish *S. stercoralis* infected patients from non-infected individuals (p<0.0001), with an AUC higher than 0.8, which represents a good accuracy of diagnostic test. Among all clones, C9 showed the best results for AUC (0.871), with sensitivity of 87.5%, specificity of 80% and diagnostic accuracy of 82.5%, followed by clones C10 and D3, which also had lower cross-reactivity with serum samples from patients with other parasites. The high LR+ value for C9 was also very good and represents a high probability of a true positive strongyloidiasis diagnosis, while low LR- is suitable in ruling out the disease. Our results are comparable to the use of crude extracts of *S. venezuelensis* in serological detection of S. stercoralis infection (90%) presented elsewhere [18] with the advantage of being recombinant clones that can be produced in large scale in the supernatant of E. coli culture.

Recent advances in immunology of infectious diseases suggest that efficient serodiagnosis requires high antigenicity of immunodominant epitopes to enable antibodies to be developed in most, if not all, infected individuals, and the antigen should contain the epitope or its substitutes. The introduction of assays based on recombinant antigens that can be produced in large quantities offers attractive alternatives to the use of crude antigen, which requires the maintenance of laboratory animals with chronic infections or stool collections from infected individuals for antigen production [50]. Peptide sequences selected from Phage display libraries against circulating antibodies to microorganisms and parasites have been described as mimotopes for hepatitis B virus [50], HIV-1 [51], and neurocysticercosis [35], [53], [54].

Other investigations have used a 31-kDa recombinant antigen (termed NIE) with significant accuracy in the serodiagnosis of strongyloidiasis [25], [26]; however those studies did evaluate cross-reactivity with a very restricted group, only consisted of filaria-infected patients. Studies of cross-reactivity in groups of patients with greater heterogeneity of parasitic infections, especially in endemic regions, must be peformed and it is essential to assert on any specific antigen or diagnostic test. We have conducted a comprehensive approach regarding the cross-reactivity, and tested all selected clones in a more diverse group of patients with various intestinal parasites, and surprisingly we have observed a very low cross-reactivity, considering the endemic region where parasitic diseases are highly prevalent. For this reason, cross-reactions among serological tests for strongyloidiasis may occur, and are usually found against schistosomes and hookworms [5], [21], [22].

The restricted sample size used for *Trichuris* sp. and *Giardia lamblia* in our cross-reactivity assays can be considered as a weakness of this study; however, all species taken into account, except the aforementioned ones, are known to present serological cross-reactivity with sera from patients with *S. stercoralis* infection [5], [21], [22]. The partial cross-reactions of those two species were not expected due to their different phylogenetic origins. The cross-reactivity may be explained by a possible reaction with the phage particle, suggesting that the fused peptide must be synthesized and tested, or those patients may have been exposed to *S. stercoralis* in some previous time, maintaining an immunological memory. The same argument can also be used for those reactive healthy individuals, regarded as false positives. More studies are being conducted in order to better explain these cases, discriminating the active infection with *S. stercoralis* from suspected cases or false positives serological results, and to differentiate recent from past infections [47].

Thus, the low cross-reactivity observed for the C9 clone, with satisfactory specificity (80%), suggest its use as a promising antigen in the serodiagnosis of strongyloidiasis, which may also include the C10 clone (77.5%). Furthermore, these novel antigens may be routinely used in large screening programs, contributing directly to the control and prevention of the disease due to the easy and direct antigen production coupled to a simple immunoassay. Differently from techniques that are not easy to attain in simple laboratory settings, specifically the use of the 31-kDa recombinant antigen, the specific luciferase-antigen fusion construct, and mammalian cultures [25],[26], we have developed a very simple assay with simple production of the antigen through bacterial culture and its direct application in a phage-ELISA system.

In the competitive Phage-ELISA assay, we observed a partial reduction of the reactivity of clones in a dose-dependent manner, allowing us to affirm that the phage clones selected by phage display technology may mimic epitopes present in the total antigenic extract from *S. venezuelensis*, which is a heterologous antigen of *S. stercoralis.* Since *S. venezuelensis* has shared many epitopes with *S. stercoralis* and in great amounts, it was used as an alternative antigen in strongyloidiasis diagnosis. In other words, the selected phage-displayed peptides present a great potential to be used in future vaccine trials, which is already under investigation in order to demonstrate if these epitopes can induce production of neutralizing antibodies anti-*S. stercoralis*.

In conclusion, our novel biomarkers that are capable of detecting specific circulating antibodies in human strongyloidiasis with reasonable sensitivity and specificity, may be promptly produced through bacterial cultures, and used in several assays, such as gel agglutination tests, electrochemical sensors, magnetic hybrid capture ELISA, and many others. But the most important is the possibility of its direct application in simple Phage-ELISA tests, which may be used as a screening platform to detect and control human strongyloidiasis.

## Supporting Information

Checklist S1 STARD checklist.
(DOC)
